# Ophthalmic Manifestations of COVID-19: A Retrospective Study on Prevalence, Characteristics, and Clinical Implications

**DOI:** 10.7759/cureus.59177

**Published:** 2024-04-27

**Authors:** Monika Patel, Rohankumar Gandhi, Niketkumar Satapara, Dhruvam L Babaria, Rishita Bakulbhai Vala, Yogesh Murugan

**Affiliations:** 1 Community and Family Medicine, Guru Gobind Singh Government Hospital, Jamnagar, IND; 2 Community and Family Medicine, Shri M.P. Shah Medical college, Jamnagar, IND; 3 Community and Family Medicine, Shri M.P. Shah Medical College, Jamnagar, IND; 4 Internal Medicine, Shri M.P. Shah Medical College, Jamnagar, IND; 5 Internal Medicine, Urban Primary Health Care, Jamnagar Municipal Corporation, Jamnagar, IND; 6 Family Medicine, Guru Gobind Singh Government Hospital, Jamnagar, IND

**Keywords:** large data analysis. retrospective study design, dry eye disorder, viral conjunctivitis, ophthalmic manifestations, sars-cov-2, covid-19 india

## Abstract

Background: The coronavirus disease 2019 (COVID-19) pandemic, caused by the SARS-CoV-2 virus, has had far-reaching implications beyond the respiratory system. Mounting evidence suggests that COVID-19 can impact various organ systems, including the eyes. This study aimed to elucidate the prevalence, characteristics, and clinical implications of ophthalmic manifestations in patients diagnosed with COVID-19.

Methods: This retrospective study analyzed data from electronic medical records of adult patients hospitalized with COVID-19 between March 1, 2020, and December 31, 2020, at a large tertiary academic medical center. Ophthalmic manifestations, including conjunctivitis, dry eye symptoms, ocular pain, epiphora, ocular redness, and visual disturbances, were identified and examined for their prevalence, onset, duration, and associations with COVID-19 severity and systemic symptoms.

Results: Among 342 patients included in the study, 106 (31.0%) experienced at least one ophthalmic manifestation during their COVID-19 illness. Conjunctivitis was the most common manifestation in 62 patients (18.1%), followed by dry eye symptoms in 38 patients (11.1%), ocular pain/discomfort in 27 patients (7.9%), epiphora in 19 patients (5.6%), ocular redness in 14 patients (4.1%), and visual disturbances in nine patients (2.6%). Ophthalmic manifestations were significantly associated with severe COVID-19 illness (42.9% vs. 26.7%, p = 0.003) and the presence of systemic symptoms such as fever, cough, and dyspnea. The median time of onset was six days, and the median duration was 10 days.

Conclusions: Ophthalmic manifestations are prevalent in COVID-19 patients, particularly in those with severe illness and systemic symptoms. The study highlights the importance of recognizing and managing ocular symptoms in affected individuals and underscores the multifaceted nature of COVID-19, necessitating interdisciplinary collaboration for comprehensive patient care.

## Introduction

The coronavirus disease (COVID-19) pandemic, stemming from the novel coronavirus severe acute respiratory syndrome coronavirus 2 (SARS-CoV-2), has presented unprecedented challenges to global public health systems since its onset in late 2019. While primarily acknowledged as a respiratory ailment, mounting evidence suggests that COVID-19 can impact various organ systems, including the eyes. The attention to ophthalmic manifestations linked with COVID-19 has been increasing due to their potential implications for disease prognosis and management [1].

While the initial emphasis was on the respiratory system, other bodily systems are now gaining recognition. Specifically, the eyes have become a focal point of study. Early research indicates that COVID-19 may be transmitted through the lacrimal ducts of the eye [2]. SARS-CoV-2 has been found to bind to the angiotensin-converting enzyme 2 (ACE2) receptor and transmembrane protease serine 2 (TMPRSS2), which are also present in eye tissue, suggesting a potential entry route via the eyes [3,4]. Apart from being a possible transmission vector, the eyes have also been the subject of research concerning the manifestations and complications of COVID-19. Initial observations have documented various symptoms such as dry eye, foreign body sensation, itching, conjunctivitis, and changes in visual acuity. Most reported cases were transient, with ocular abnormalities resolving following recovery from COVID-19 [5].

Despite increasing attention, there remains an incomplete understanding of the ocular manifestations and complications associated with COVID-19 infection. Challenges are compounded by the uncertain prevalence of asymptomatic COVID-19 carriers who are unaware and not receiving medical care [6].

By elucidating the epidemiology and clinical characteristics of ophthalmic manifestations in COVID-19, our study can contribute to the broader understanding of the disease's multisystemic nature. Furthermore, it underscores the importance of interdisciplinary collaboration between ophthalmologists, infectious disease specialists, and primary care providers in managing COVID-19 patients comprehensively.

In this retrospective study conducted at a tertiary care hospital in Gujarat, we aimed to elucidate the prevalence, characteristics, and clinical implications of ophthalmic manifestations in patients diagnosed with COVID-19. Our study comprehensively examined various ophthalmic manifestations, including conjunctivitis, dry eye symptoms, ocular pain, epiphora, ocular redness, and visual disturbances, among others. We investigated their prevalence, onset, duration, and associations with the severity of COVID-19 illness and systemic symptoms.

## Materials and methods

Study design and setting

This was a retrospective study conducted at a large tertiary academic medical center (Guru Gobind Singh Government Hospital, Jamnagar, India). We analyzed data from the hospital records of all adult patients ≥18 years old who were hospitalized with COVID-19 between March 1, 2020, and December 31, 2020.

Study population

The study included patients aged 18 years or older who were diagnosed with COVID-19 between March 1, 2020, and December 31, 2020. COVID-19 diagnosis was confirmed by a positive result on a reverse transcriptase-polymerase chain reaction (RT-PCR) test for SARS-CoV-2 from a nasopharyngeal or oropharyngeal swab.

Sampling strategy

Patients included in this study were selected using a consecutive sampling method. All adult patients aged 18 years or older who were hospitalized with COVID-19 between March 1, 2020, and December 31, 2020, at the large tertiary academic medical center were considered for inclusion. This approach ensured that all eligible patients during the specified timeframe were included, without any predetermined selection criteria.

Exclusion criteria

Patients with pre-existing ophthalmic conditions or those who had undergone recent ophthalmic procedures were excluded from the study to mitigate potential confounding factors. Additionally, patients with incomplete medical records or missing key data points relevant to the study objectives were excluded to ensure the reliability and validity of the findings. These exclusion criteria were applied uniformly to maintain consistency in the patient selection process and minimize bias in the study results.

Data collection

Electronic medical records (EMRs) of patients with confirmed COVID-19 were reviewed for demographic information, medical history, clinical characteristics, laboratory findings, and ophthalmic manifestations during their illness.

Ophthalmic manifestations were identified based on documented symptoms, signs, and findings in the EMRs, including conjunctivitis (unilateral or bilateral), dry eye symptoms (burning, foreign body sensation, blurred vision), ocular pain or discomfort, epiphora (excessive tearing), ocular redness, and visual disturbances (blurred vision, scotoma)

Other data collected included the time of onset of ophthalmic manifestations relative to the onset of COVID-19 symptoms, the duration of ophthalmic manifestations, and the severity of COVID-19 illness (mild, moderate, severe, or critical) based on the combination of clinical variables (symptoms, physical findings, radiographic and laboratory values)

Quality measures

To ensure the integrity and reliability of the data collected, stringent quality control measures were implemented throughout the study. A standardized data extraction protocol was developed, providing detailed instructions for identifying and recording pertinent information from EMRs. This protocol was adhered to consistently by trained research personnel, who underwent thorough training sessions to familiarize themselves with the study procedures. Additionally, inter-rater reliability checks were performed regularly, wherein a subset of patient records was independently reviewed by multiple researchers to assess the consistency of data extraction. Any discrepancies or inconsistencies were resolved through consensus discussions or review by senior investigators. Calibration exercises further reinforced adherence to the protocol and minimized variability in data interpretation and recording. Periodic audits of the data extraction process were conducted by the principal investigators to monitor compliance with the protocol and identify any deviations necessitating corrective action. Furthermore, data validation procedures were employed to verify the accuracy and completeness of extracted data by cross-referencing entries with original source documents in the EMRs. These comprehensive quality control measures were instrumental in upholding the reliability, validity, and integrity of the data collected, thereby bolstering the credibility of the study findings.

Data analysis

Descriptive statistics were used to summarize the demographic and clinical characteristics of the study population, as well as the prevalence and types of ophthalmic manifestations. Categorical variables were reported as frequencies and percentages, while continuous variables were reported as means with standard deviations or medians with interquartile ranges, as appropriate. The association between ophthalmic manifestations and COVID-19 severity, as well as the presence of systemic symptoms (fever, cough, dyspnea), was analyzed using Chi-square or Fisher's exact tests for categorical variables, as appropriate. The time of onset and duration of ophthalmic manifestations were reported as medians with interquartile ranges. All statistical analyses were performed using IBM SPSS Statistics for Windows, Version 26 (Released 2019; IBM Corp., Armonk, New York, United States), with a p-value of less than 0.05 considered statistically significant.

## Results

Table 1 presents the demographic and clinical characteristics of the study population. Out of the total 342 patients included in the study, the mean age was 48.7 years, ranging from 18 to 84 years. The majority of patients were male, with 197 (57.6%) being male. The most common comorbidities were hypertension, present in 109 patients (31.9%), diabetes mellitus in 77 patients (22.5%), and cardiovascular disease in 49 patients (14.3%).

**Table 1:** Demographic and Clinical Characteristics

Characteristic	Frequencies (%)
Total Patients	342 (100%)
Mean Age (range)	48.7 years (18-84 years)
Male Gender	197 (57.6%)
Hypertension	109 (31.9%)
Diabetes Mellitus	77 (22.5%)
Cardiovascular Disease	49 (14.3%)

Table 2 shows the prevalence of ophthalmic manifestations among the study population. Overall, 106 patients (31.0%) experienced at least one ophthalmic manifestation during their COVID-19 illness. The most common ophthalmic manifestation was conjunctivitis, observed in 62 patients (18.1%), with 28 patients (45.2%) having unilateral conjunctivitis and 34 patients (54.8%) having bilateral conjunctivitis. Additionally, 38 patients (11.1%) experienced dry eye symptoms, 27 patients (7.9%) had ocular pain or discomfort, 19 patients (5.6%) reported epiphora (excessive tearing), 14 patients (4.1%) had ocular redness, and nine patients (2.6%) experienced visual disturbances.

**Table 2:** Prevalence of Ophthalmic Manifestations

Ophthalmic Manifestation	Frequency (%)
Any Ophthalmic Manifestation	106 (31.0%)
Conjunctivitis	62 (18.1%)
Unilateral	28 (45.2%)
Bilateral	34 (54.8%)
Dry Eye Symptoms	38 (11.1%)
Ocular Pain/Discomfort	27 (7.9%)
Epiphora	19 (5.6%)
Ocular Redness	14 (4.1%)
Visual Disturbances	9 (2.6%)

Table 3 demonstrates the association between ophthalmic manifestations and the severity of COVID-19 illness. Patients with severe COVID-19, requiring hospitalization or intensive care unit (ICU) admission, had a significantly higher prevalence of ophthalmic manifestations compared to those with mild or moderate illness (42.9% vs. 26.7%, p = 0.003). The odds ratio for developing ophthalmic manifestations in severe COVID-19 cases was 2.078 (95% CI: 1.27-3.38), indicating that patients with severe illness were approximately twice as likely to experience ophthalmic manifestations.

**Table 3:** Association between Ophthalmic Manifestations and COVID-19 Severity *Statistically significant (p < 0.05) COVID-19: Coronavirus disease 2019

COVID-19 Severity	Ophthalmic Manifestations Present n(%)	Ophthalmic Manifestations Absent n(%)	OR	p-value
Severe (Hospitalized/ICU)	42 (42.9%)	56 (57.1%)	2.08	0.003*
Mild/Moderate	64 (26.7%)	176 (73.3%)		

Table 4 examines the association between ophthalmic manifestations and the presence of systemic symptoms. Patients who experienced fever had a significantly higher prevalence of ophthalmic manifestations compared to those without fever (38.7% vs. 61.3%, p = 0.007). Similarly, patients with cough (36.4% vs. 63.6%, p = 0.049) and dyspnea (45.8% vs. 54.2%, p = 0.002) were more likely to develop ophthalmic manifestations than those without these symptoms.

**Table 4:** Association between Ophthalmic Manifestations and Systemic Symptoms *Statistically significant (p < 0.05)

Systemic Symptom	Ophthalmic Manifestations Present n(%)	Ophthalmic Manifestations Absent n(%)	p-value
Fever	48 (38.7%)	76 (61.3%)	0.007*
Cough	47 (36.4%)	82 (63.6%)	0.049*
Dyspnea	33 (45.8%)	39 (54.2%)	0.002*

Table 5 presents data on the time of onset and resolution of ophthalmic manifestations. The majority of patients (72.6%) experienced ophthalmic manifestations within the first two weeks of COVID-19 symptom onset, with a median time of onset of six days (interquartile range: 3-10 days). The median duration of ophthalmic manifestations was 10 days (interquartile range: 5-16 days) among the subset of 84 patients with follow-up data. However, three patients (3.6%) experienced persistent visual disturbances, which did not resolve during the study period.

**Table 5:** Time of Onset and Resolution of Ophthalmic Manifestations IQR: Interquartile Range

Characteristic	Frequency (%)
Onset within the First Two Weeks	77 (72.6%)
Median Time of Onset (IQR)	6 days (3-10 days)
Median Duration (IQR) (n=84)	10 days (5-16 days)
Persistent Visual Disturbances	3 (3.6%)

These results highlight the significant prevalence of ophthalmic manifestations in patients with COVID-19, particularly in those with severe illness and systemic symptoms. The findings also provide insights into the time course and resolution of these manifestations, underscoring the importance of timely recognition and appropriate management.

## Discussion

The results of this study provide valuable insights into the prevalence, characteristics, and clinical implications of ophthalmic manifestations in patients with COVID-19. Our findings reveal that approximately one-third of the study population experienced at least one ophthalmic manifestation during their illness. Conjunctivitis emerged as the most common ophthalmic manifestation, followed by dry eye symptoms, ocular pain/discomfort, epiphora, ocular redness, and visual disturbances.

Conjunctivitis is the most common ophthalmic manifestation documented in COVID-19 patients. In a large series of cases with mild COVID-19 infection, Sindhuja et al. reported that 11/127 (8.66%) patients had conjunctivitis [7]. Similarly, a meta-analysis by Ling et al. found that the pooled prevalence of conjunctivitis in COVID-19 patients was 3.8% (95% CI: 2.1-6.3%). However, the prevalence rates vary across studies, likely due to differences in study populations, disease severity, and diagnostic criteria [8].

Our study observed a significant association between the severity of COVID-19 illness and the presence of ophthalmic manifestations. Patients with severe illness, necessitating hospitalization or ICU admission, were approximately twice as likely to experience ophthalmic manifestations compared to those with mild or moderate illness. This aligns with previous research by Aggarwal et al. and Ulhaq and Soraya, who reported a higher prevalence of ophthalmic manifestations in severe COVID-19 cases [9,10]. Such findings underscore the potential utility of ophthalmic manifestations as indicators of disease severity and warrant close monitoring of ocular symptoms in critically ill patients. Inomata et al. suggest that ocular surface manifestations may be a prodromal sign of more severe COVID-19 illness, highlighting the importance of early recognition and intervention [11].

Furthermore, our study identified a significant association between the presence of systemic symptoms, such as fever, cough, and dyspnea, and the development of ophthalmic manifestations. Patients with these systemic symptoms were more likely to experience ocular manifestations compared to those without such symptoms. This observation aligns with the findings of Korompoki et al., who reported that ocular manifestations were more prevalent in COVID-19 patients with systemic symptoms [12]. The association between systemic symptoms and ocular involvement suggests a potential systemic inflammatory response contributing to ocular manifestations in COVID-19, as proposed by Danthuluri and Grant [13].

Regarding the timing of ophthalmic manifestations, our study found that the majority of patients experienced ocular symptoms within the first two weeks of COVID-19 symptom onset, with a median time of onset of six days. This is consistent with findings from Mangana et al. and Chen et al., who reported a broad timeline for the onset of ocular symptoms ranging from the initial presentation of COVID-19 to several weeks after symptom onset [14,15]. Bal et al. suggest that ocular manifestations can appear at any stage of the disease, with conjunctivitis often being an early sign, while later ocular pathology may be linked to more severe COVID-19. Although the evidence for direct transmission through eye mucosa is currently lacking, early detection of COVID-related ocular symptoms could potentially limit disease spread [16,17].

However, it is worth noting that some patients experienced persistent visual disturbances, highlighting the need for long-term monitoring and management of ocular sequelae in COVID-19 survivors. Mendelson et al. emphasize the potential for long-lasting complications, termed "long-COVID," necessitating ongoing care and rehabilitation for affected individuals [17].

Overall, our study contributes to the growing body of evidence on the ophthalmic manifestations of COVID-19, emphasizing the importance of recognizing and managing ocular symptoms in affected individuals. The observed associations with disease severity and systemic symptoms underscore the multifaceted nature of COVID-19 and the potential systemic implications of ocular involvement. Moving forward, collaborative efforts between ophthalmologists, infectious disease specialists, and primary care providers are essential for the comprehensive management of COVID-19 patients, focusing on early detection and appropriate treatment of ophthalmic manifestations.

Overall, our study contributes to the growing body of evidence on the ophthalmic manifestations of COVID-19, emphasizing the importance of recognizing and managing ocular symptoms in affected individuals. The observed associations with disease severity and systemic symptoms underscore the multifaceted nature of COVID-19 and the potential systemic implications of ocular involvement. Moving forward, collaborative efforts between ophthalmologists, infectious disease specialists, and primary care providers are essential for the comprehensive management of COVID-19 patients, focusing on early detection and appropriate treatment of ophthalmic manifestations.

Strengths

This study's strength lies in its comprehensive examination of various ophthalmic manifestations in COVID-19 patients, including conjunctivitis, dry eye symptoms, ocular pain, epiphora, ocular redness, and visual disturbances. By investigating the prevalence, onset, duration, and associations of these manifestations with disease severity and systemic symptoms, the study provides a holistic understanding of the ocular implications of COVID-19. The large sample size of 342 patients and the inclusion of hospitalized cases across a broad severity spectrum further enhance the robustness and generalizability of the findings. Additionally, the study's focus on the temporal aspects of ophthalmic manifestations, such as the time of onset and duration, contributes valuable insights into the clinical course and management of these symptoms.

Limitations

One limitation of the study is its retrospective design, which relies on the accuracy and completeness of information documented in electronic medical records. There is a possibility of underreporting or missing data, particularly for milder ophthalmic manifestations that may have been overlooked or not documented thoroughly. Furthermore, the study did not include a control group of non-COVID-19 patients, which could have provided a reference point for comparing the prevalence of ophthalmic manifestations in the general population. The study's single-center design may also limit the generalizability of the findings to other geographic regions or healthcare settings with different patient populations or clinical practices. Additionally, the study did not investigate the potential mechanisms or pathophysiological processes underlying the observed ophthalmic manifestations, which could be an area for future research.

## Conclusions

This retrospective study provides valuable insights into the prevalence, characteristics, and clinical implications of ophthalmic manifestations in patients with COVID-19. The findings underscore the significant prevalence of ocular symptoms, particularly in severe cases of COVID-19, and highlight the importance of interdisciplinary collaboration for comprehensive patient care. While the study has strengths in its comprehensive assessment and large sample size, limitations such as its single-center design and potential biases in data collection should be considered. Moving forward, further research is warranted to validate these findings and explore the long-term implications of ocular involvement in COVID-19 survivors.
